# Sustainability of the streamlined ART (START-ART) implementation intervention strategy among ART-eligible adult patients in HIV clinics in public health centers in Uganda: a mixed methods study

**DOI:** 10.1186/s43058-020-00025-5

**Published:** 2020-03-30

**Authors:** Richard Katuramu, Moses R. Kamya, Naome Sanyu, Mari Armstrong-Hough, Fred C. Semitala

**Affiliations:** 1 Department of Internal Medicine, Makerere University College of Health Sciences, P.O. Box 7072, Kampala, Uganda; 2 Infectious Diseases Research Collaboration, Kampala, Uganda; 3 Makerere University Joint AIDS Program, Kampala, Uganda; 4 New York University, New York, USA

**Keywords:** Barriers, Facilitators, START-ART, Sustainability, HIV, Implementation

## Abstract

**Background:**

Despite increasing access to antiretroviral therapy (ART), the proportion of eligible patients initiated on treatment remains suboptimal. Only 64.6% of the people living with HIV (PLHIV) globally were initiated on ART by June 2019. The streamlined ART (START-ART) implementation study was based on the PRECEDE model, which suggests that “predisposing, enabling, and reinforcing” factors are needed to create behavior change. START-ART increased ART initiation within 2 weeks of eligibility by 42%. However, the gains from some implementation interventions erode over time. We evaluated facilitators and barriers to sustainability of this streamlined ART initiation in the year following the implementation period.

**Methods:**

We designed a mixed-methods explanatory sequential study to examine the sustainability of START-ART implementation. Quantitative component consisted of cross-sectional patient chart reviews of routinely collected data; qualitative component consisted of key informant interviews of health workers in START-ART facilities 2 years after conclusion of the implementation period. We analyzed data from 15 public health centers of Mbarara district, where the START-ART implementation was carried out. We included PLHIV aged > 18 years who initiated ART from June 2013 to July 2016. The START-ART implementation took place from June 2013 to June 2015 while the sustainability period was from August 2015 to July 2016.

**Results:**

A total of 863 ART-eligible patients were sampled. The median CD4 count was 348 cells/ml (IQR 215–450). During the intervention, 338 (77.4%) eligible patients initiated on ART within 2 weeks compared with 375 (88.2%) during the sustainability period (risk difference 10.8%; 95% CI 5.9–15.8%). In 14 of the 15 health centers, the intervention was sustained. During key informant interviews, rapid ART initiation sustainability was attributed to counseling skills that were obtained during intervention and availability of point-of-care (POC) CD4 PIMA machine. Failure to sustain the intervention was attributed to three specific barriers: lack of training after the intervention, transfer of trained staff to other health facilities, and shortage of supplies like cartridges for POC CD4 PIMA machine.

**Conclusion:**

Rapid ART initiation was sustained in most health centers. Skills acquired during the intervention and functional POC CD4 machine facilitated while staff transfers and irregular laboratory supplies were barriers to sustainability of rapid ART initiation.

Contributions to the literature

- Implementation science interventions should be integrated into the existing health delivery system as an essential component for sustainability.
- Future translational research should include strategies to implement large-scale facility-based initiatives involving all the partners in HIV care with the aim of attaining sustainability after successful implementation.
- Strategies to overcome the physical and psychological challenges to facilitate successful uptake of interventions should be utilized to implement interventions.

## Introduction

Despite increasing access to antiretroviral therapy (ART), the proportion of eligible patients initiated on treatment remains suboptimal [1, 2]. The 90-90-90 targets aim for 90% of people living with HIV (PLHIV) to know their HIV status, 90% of PLHIV to be on ART, and 90% of patients on ART to attain virological suppression by 2020 [3]. Globally, it is estimated only 64.6% of the PLHIV were initiated on ART by June of 2019 [4]. The use of ART dramatically reduces AIDS-related illnesses and deaths among PLHIV, especially benefitting those who initiate earlier at higher CD4 counts [5, 6]. Moreover, ART use reduces the risk of HIV transmission [7]. In 2013, increased ART use was estimated to have averted 6.3 million AIDS-related deaths worldwide [8]*.* However, delayed initiation of ART among PLHIV who have presented to care in Sub-Saharan Africa (SSA) are common. A systematic review showed the median time to ART initiation of PLHIV who presented in care is longer than a month [9]. As a result of this delay, an estimated 30 to 35 deaths occur per 100 person years among PLHIV in care before they are initiated on ART in SSA [10, 11]. It is also estimated that mortality increases by 34% for every 10-week delay in ART initiation among patients entering care [9]. In addition, delayed ART initiation is associated with increased risk of loss to follow-up prior to treatment initiation [9].

We previously carried out and evaluated a multi-component implementation intervention in the streamlined ART (START-ART) implementation study (ClinicalTrials.gov, number NCT01810289) in Uganda which has been reported elsewhere [12, 13]. The implementation intervention consisted of provider education about the importance of rapid ART initiation, introduction of novel technology of testing for CD4 counts using point-of-care CD4 PIMA machine, and provision of reinforcing feedback to the health care providers, to increase the rapidity and completeness of ART initiation [12]. In the 2 years (April 2013 to June 2015) of implementing the START-ART intervention, the proportion of PLHIV initiated on ART within 14 days increased by 42% from 37.7% in the control group to 79.6% in the intervention group [12]. The START-ART implementation study was carried out with a strategy to be sustained after the end of its implementation.

To prepare health care workers and district administrators for transition from the study team to the sustainability period, a joint meeting with the in-charges of participating health centers, district health officials, and district implementing partner was held 6 months before the end of the intervention period. Stakeholders were informed that all the components of the intervention would transition to management by the Ministry of Health and its implementing partners, so that rapid ART initiation could continue within the health centers beyond the study period. The transition involved handing over the POC PIMA machines to district health officials and district implementing partners and a commitment to continuation of rapid ART initiation in all participating health facilities. The study team met with health care workers at all study sites to inform them of the transition plan.

The objective of the present study was to evaluate the sustainability of the START-ART implementation study, here defined as the continuation of the initiation of ART within 14 days of documented eligibility, during the year following the end of the START-ART implementation study. We further aimed to identify the facilitators and barriers to the sustainability of rapid ART initiation beyond the study period among public health centers that participated in the START-ART implementation study.

## Methods

### Study design and setting

We used a mixed-methods explanatory sequential research design that involved both quantitative and qualitative methods. The quantitative study was a cross-sectional design while the qualitative study involved key informant interviews.

The study was conducted in 15 of the 20 public health centers where the START-ART intervention was carried out. These included three health center IVs (HCIV) and 12 health center IIIs (HCIII). The remaining five health centers were excluded from the sampling frame because they are centers of excellence (COE) for HIV care; therefore, findings from these centers could not easily be generalized to other public health centers in Uganda.

All the selected 15 health centers that took part in this study are found in Mbarara district, which is located in the southwestern part of Uganda, approximately 265 km from Kampala, Uganda’s capital city. These health centers are operated by the Ministry of Health and supported by the Makerere University Joint AIDS Program (MJAP), a program supported by the President’s Emergency Plan for AIDS Relief (PEPFAR). All these health centers offer both inpatient and outpatient medical and surgical services. Some have their HIV clinics running from Monday to Friday between 8.00 am and 5.00 pm, while others operate once or twice a week.

### The START-ART implementation study

Briefly, the START-ART implementation study was a cluster-randomized, stepped-wedge implementation study conducted between April 2013 and June 2015 in 20 public health centers that offered ART to PLHIV. The study included all treatment-naïve HIV-infected adults who met the criteria for ART initiation as recommended by the Ugandan HIV guidelines during the study period [14]. It was based on the PRECEDE model, which suggests that “predisposing, enabling, and reinforcing” factors are needed to create behavior change among health care workers [15]. As a predisposing factor, the START-ART study engaged HIV care opinion leaders to lead interactive training sessions. In these sessions, recent scientific evidence regarding the benefits of rapid initiation of ART [16, 17], and the risks of delayed ART initiation was conveyed to frontline HIV care providers. For the enabling factor, we introduced a point-of-care (Pima™) CD4 cell count test machine at each of the study clinics. The PIMA delivered an absolute count of T helper cells from either a finger stick or venous whole blood sample within 20 min, allowing determination of treatment eligibility on the same day as presentation [18]. As a reinforcing factor, twice every year, the study team provided feedback to the leaders (“in-charges”) as well as HIV care providers at each of the participating health centers. The feedback involved presentation of the health center ART initiation rates as compared with other health centers to motivate health care providers. Further details of the design, methods, and results of the START-ART implementation study have been described elsewhere [12]. Ethical approval of the study was granted by the School of Medicine Research and Ethics committee at the Makerere University.

### Quantitative study

#### Study participants

These were PLHIV who initiated ART during the START-ART *intervention period* (19 June 2013 to 30 June 2015) and *sustainability period* (1 August 2015 to 31 July 2016.) We defined the *intervention period* as the period when the START-ART implementation intervention was actively applied. We defined the *sustainability period* as the period beginning a full 2 months after intervention activities ceased. We defined *sustainment* as having maintained or improved the proportion of eligible patients initiated on ART during the intervention period throughout the sustainability period. For a health center to qualify to have sustained the intervention, the proportion of PLHIV who were initiated onto ART during the sustainability period should be equal to or more than the proportion of PLHIV who initiated ART treatment during the START-ART intervention, or less by a difference of not more 10%.

#### Sample size estimation

We determined a sample size based on a 10% difference in the risk of ART initiation at or before 14 days of eligibility in both the sustainability and intervention groups, with *α* of 0.05, power of 90%, and a ratio of intervention and sustainability of 1:1. We added 80 participants (10%) to our calculated sample of 796 study participants to adjust for missing ART charts. Thus, the final sample comprised of 876 patient records [19].

#### Sampling methods

Patients’ ART treatment charts were the sampling units in both the intervention and sustainability periods. All information that was recorded in the treatment charts was obtained electronically. Using the patients’ date of ART eligibility, patients who were eligible to take part in the study were selected. Because there are differences in the size and numbers of patients enrolled in each health center, we used a systematic sampling method basing on probability proportional to size, in which each health center contributed a pre-determined sample size. This depended on the percentage each center contributed to the total number of patients who were eligible for this study in all the health centers. A random patient identification number (ID) was selected by fish bowl method. In order to determine the interval at which we were to select by ID, we divided the total number of PLHIV in each health center by the sample we were to obtain from that same health center, arriving at an interval of four in all health centers. We therefore selected every four ID until the sample size from that specific health center was reached. If a file for an eligible patient was not found, we selected the next eligible patient whose ID followed in chronological order.

#### Study variables

Data were collected on socio-demographic characteristics, including age, gender, religion, marital status, occupation, level of health center, and sex. The medical and laboratory history variables collected were as follows: date of testing for HIV status, use of cotrimoxazole prophylaxis, CD4 test completion and date, WHO stage, date eligible for ART, date of ART initiation, ART regimen, and co-morbidities. These were entered into an ART electronic database, which was designed during the initial period of study. Data were captured for all patients eligible for ART and all who initiated on ART during the period of study. The primary outcome was time in days to treatment initiation among ART-eligible patients.

#### Study procedures

For the START-ART intervention, the variables were obtained from the START-ART data base which was collected during the intervention period. For the sustainability period, the variables of interest were collected from the medical ART charts at the 15 selected health facilities. These were collected using a standardized, pre-tested questionnaire. They were then entered into an Epi-data database that was specifically designed for this study. We selected the files with a systematic sampling method using the patients’ identification numbers from the ART registry until the number selected from each health center was attained.

#### Statistical analysis

Patient characteristics for continuous variables were described by mean and standard deviation for normally distributed data, while median and interquartile ranges were used for the observations that were not normally distributed. Categorical variables were described using frequencies and percentages. Student’s *t* test was used for bivariate analysis of continuous data, and the chi-squared test was used for categorical variables. *p* value less than 0.05 were considered statistically significant.

Our study end point was ART initiation within 14 days after first date of eligibility during the study period. The primary outcome was treated as binary. We assessed patients as either initiating ART by 14 days or not. We used a binomial regression model for the analysis of assess whether baseline demographics namely sex, age, level of health center, CD4 cells count at eligibility, TB status at eligibility, pregnancy status at eligibility, WHO clinical stage at eligibility, and location of health center were associated with ART initiation within 14 days after the first date of eligibility comparing between the START-ART and sustainability periods. The measure of effect was risk differences and all the *p* values were based on an alpha of 5% and all tests two-tailed. The measure of effect was risk differences, and all tests were two-tailed.

We used a stacked bar graph to examine the secular trends from the START-ART intervention and the sustainability periods.

The findings have been reported using the Strengthening the Reporting of Observational Studies in Epidemiology (STOBE) statement: guidelines for reporting observational studies [20]. Analysis was done using STATA version 12.0.

### Qualitative study

#### Study population

We assessed facilitators and barriers to continuation of the components of the START-ART intervention using key informant (KI) semi-structured interviews with health care workers involved in care of HIV-positive patients from the participating health centers. These included clinic in charges/clinician, nurses/counselors, laboratory technicians, and PLHIV peer educators.

#### Sampling for key informant interviews

After analyzing data from the quantitative study, two health facilities at opposite ends of the spectrum (sustained versus not sustained) were selected at random. We randomly selected one health facility where the START-ART intervention was sustained to explore factors that facilitated the sustainability of the START-ART intervention in these health facilities. We also selected one health center in which the START-ART intervention was not sustained to explore the barriers to sustainability of the intervention. Both were health center III level. Each provides outpatient and inpatient care for non-severe illnesses and operates an HIV clinic 1 day per week. On that day, they receive on average about 50–60 patients.

In each selected health center, we conducted key informant interviews with a purposive selection of four health care workers, including a clinic in-charge who also acted as the clinician, one nurse or ART counselor, one laboratory technician, and one peer educator.

#### Data collection for key informant interviews

An interview guide with open-ended questions was used to explore health providers’ views about how the START-ART intervention influenced their clinical care after the study ended, as well as their understandings of the guidelines for ART initiation, including both current guidelines used by the clinic and those that were used in the START-ART study. We also explored health providers’ approach to ART initiation in their clinic, including the time required to initiate eligible patients on ART and providers’ ability to recall the components of the START-ART intervention.

These key informant interviews were conducted by a trained research assistant (NS) between February and March 2018, approximately one and half years after the end of the sustainability period. The interviews were conducted using semi-structured interview guides to explore facilitators and perceived barriers to sustainability START-ART intervention. Pre-testing was conducted in a nearby health facility which was not part of the study health facilities, but which had similar settings to the study facilities before the commencement of the study. At least one health worker in each cadre (clinic in-charge, nurses or ART counselor, laboratory technician, and peer educator) involved in ART clinic in the selected health facility was selected for interviews.

All interviews took place in private settings where other people could not hear the respondents’ answers. These interviews were conducted in English and recorded using a voice recorder. Each interview took about 15 min, after which it was transcribed by two research assistants for comparison purposes.

#### Qualitative analysis

The principal investigator (RK), who carried out this study to fulfill the award of masters of medicine in internal medicine, reviewed all transcripts and field notes in light of the study objectives. RK identified key issues and themes about facilitators and perceived barriers to accelerated ART initiation that emerged from the data. The themes were generated and coded from which the key themes that emerged were identified. These themes were then classified under the capability, opportunity or motivational components model (COM-Model) [21]. In the COM-B model, *Capability* refers to the ability of an individual to psychologically or physically engage in a behavior, *Opportunity* refers to the physical or social factors outside an individual that will influence someone’s behavior, and *Motivation* refers to the thought processes that direct a person to carry out specific behavior. The facilitators and barriers commonly reported by the providers were organized in a table under the different components of the COM-B model. Representative quotations from respondents were denoted by the cadre of the provider. The findings have been reported using Consolidated Criteria for Reporting Qualitative Research guidelines [22].

## Results

### Study enrollment

**Fig. 1:**
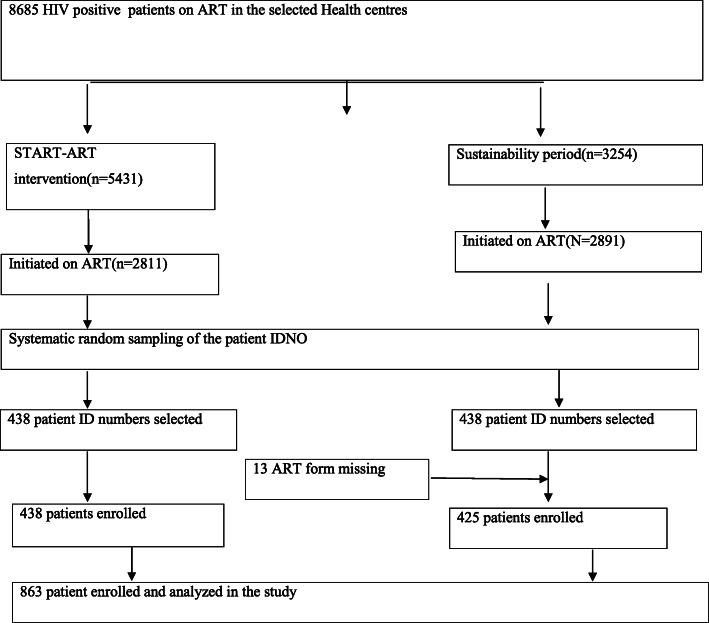
Summary of the recruitment process for the 863 enrolled patients

From 4 October 2017 to 15 February 2018, data were collected in the ART medical forms from a total of 863 patients, including 438 (50.9%) patients in the START-ART intervention study and 425 (49.1%) patients in the 1-year period following the intervention. As shown in Fig. 1, a total of 13 ART medical forms in the sustainability period were missing and excluded from the study because even after trying to look for replacements, we would end up in the next selected ID.

### Baseline characteristics of the study population

ART records for 863 patients were reviewed, 438 (50.9%) of whom presented during the START-ART implementation intervention period and the remainder of whom presented during the sustainability period. A total of 79 medical records in both the START-ART implementation period and sustainability period had missing data on the following variables: sex (0.1%), WHO clinical stage (6.7%), CD4 cell counts (14%), and pregnancy status at eligibility (3.3%). The median age for both groups was 30 years (interquartile range (IQR), 25–37). Most patients (541, 62.7%) were females, 589 (68.3%) presented in HCIII-level facilities, and 480 (55.6%) were receiving care from the rural clinics. A majority (754, 93.7%) of the PLHIV were WHO clinical stage 1 or 2 at ART eligibility and overall median CD4 cell count was 348.5cells/μl (IQR, 215-450 cells/ μl). A small number of patients (16, 1.6%) were diagnosed with TB at ART eligibility, while 165 (46.2%) females were pregnant at the time of ART initiation. Eighty-three percent (*n* = 714) initiated ART within 14 days after eligibility (Table 1).

**Table 1:** Baseline characteristics of participants during the START-ART and the sustainability period

Characteristics	During the START-ART Intervention (***N*** = 438)	Sustainability period (1 year after end of the intervention) (***N*** = 425)	***p*** value
Sex*			
Male	160 (36.5%)	161 (38.0%)	0.662
Female	278 (63.5%)	263 (62.0%)	0.009
Level of health center (HC)			
HC3	281 (64.2%)	308 (72.5%)	
HC4	157 (35.8%)	117 (17.5%)	0.744
Location of health center			
Peri-urban	192 (43.8)	191 (44.9)	
Rural	246 (56.2)	234 (55.1)	0.464
Age in years at eligibility			
≤ 35	317 (72.37)	298 (70.12)	
> 35	121 (27.63)	127 (29.90)	
Median age (IQR)	29.0 (25.0–36.0)	30.0 (25.0–38.0)	0.464
CD4 count at eligibility (cells/μl)*			
≤ 50	20 (4.56)	11 (2.6)	
> 50	376 (85.84)	335 (78.8)	
Median CD4 count (IQR)	340 (216–453)	353.5 (215–447.0)	0.017
Eligibility WHO stage*			
Stages 1 and 2	358 (81.7%)	396 (93.2%)	0.002
Stages 3 and 4	33 (7.5%)	18 (4.2%)	
Tuberculosis (TB) at ART eligibility			
TB present	2 (0.5%)	14 (3.3%)	0.688
No TB	436 (99.5%)	411 (96.7%)	
Pregnant status at ART eligibility*			
Pregnant	90 (32.4%)	75 (28.6%)	0.001
Not pregnant	188 (67.6%)	169 (64.5%)	
Time to ART initiation after becoming eligible			
Within 14 days	339 (77.4%)	375 (88.2%	
After 14 days	99 (22.6%)	50 (11.8%)	

*HC* health center

*Missing some values

More patients initiated ART from HCIII than from HCIV during the sustainability period 308 (72.5%) versus 81 (64.2%) in the START-ART implementation period (*p* = 0.009). Concurrent TB at ART eligibility was more frequent during the sustainability period, 14 (3.3%), compared to 2 (0.5%) in START-ART implementation period (*p* = 0.002). However, fewer patients had advanced HIV (WHO stages III and IV) during the sustainability period, 18 (4.2%) compared to 33 (7.5%) during the START-ART implementation (*p* = 0.017).

Table 2 shows a comparison of the proportions of patients initiated on ART within 14 days between the START-ART implementation and sustainability period.

**Table 2:** Comparing the proportion of patients initiated on ART within 14 days of eligibility between START-ART intervention and the sustainability period

	Number of patients during the intervention (***n***/***N***)	Number of patients after the intervention (***n***/***N***)	Risk ratio (95% CI)	***p*** value
Sex*				
Male	129/160 (80.60)	143/161 (88.82)	1.102 (1.003–1.210)	0.043
Female	210/278 (75.54)	231/263 (87.83)	1.163 (1.073–1.260)	0.0001
Location				
Peri-urban	149/192 (77.60)	177/191 (92.67)	1.194 (1.096–1.301)	0.0001
Rural	190/246 (77.24)	198/234 (84.62)	1.096 (1.004–1.195)	0.040
Age at eligibility (years)				
≤ 35	251/317 (79.18)	269/298 (90.27)	1.14 (1.065–1.220)	0.0001
> 35	88/121 (72.73)	106/127 (83.46)	1.15 (1.004–1.312)	0.044
ART eligibility CD4 (cells/ml)*				
≤ 50 cells/ml	18/20 (90)	9/11 (81.82)	0.909 (0.664–1.245)	0.553
> 50 cells/ml	321/418 (76.80)	295/335 (88.06)	1.147 (1.074–1.225)	0.001
Eligibility WHO stage*				
Stages 1 and 2	281/358 (78.49)	53/396 (89.14)	1.132 (1.062–1.206)	0.0001
Stages 3 and 4	29/33 (87.88)	15/18 (83.88)	0.948 (0.744–1.208)	1.208
Tuberculosis at ART eligibility				
No tuberculosis	337/436 (77.29)	363/411 (96.35)	1.143 (1.074–1.216)	0.0001
Tuberculosis	2/2 (100.0)	12/14 (85.71)	0.819 (0.661–1.014)	0.0670
Health center level				
HC3	223/281 (79.36)	266/308 (86.36)	1.088 (1.010–1.172)	0.0260
HC4	116/157 (73.89)	109/117 (93.16)	1.261 (1.135–1.401)	0.0001

^*^Missing some values

Table 2 shows a comparison of the proportions of patients initiated on ART within 14 days between the START-ART intervention and sustainability period among the different subgroups of patients. As shown in the table, there was no statistical significant difference in the proportion of patients initiated on ART within 14 days of eligibility between START-ART intervention and the sustainability period among the patients who had tuberculosis at start of ART treatment, CD4 less or equal to 50 cells/mm^3^, and eligibility WHO stages 3 and 4 (Table 2).

**Fig. 2:**
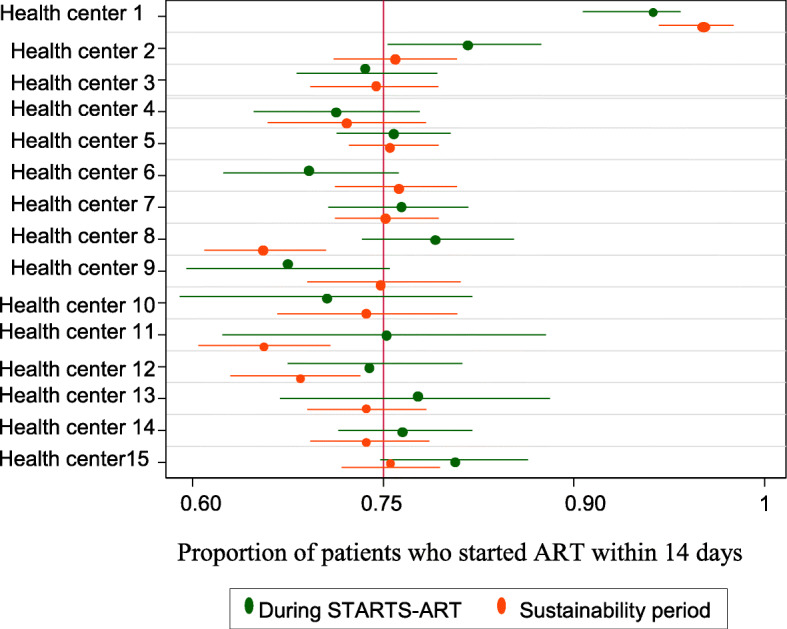
Health center analysis of ART initiation within 14 days after eligibility between the START-ART intervention and the sustainability period

An analysis of the different health centers to show ART initiation within 14 days after eligibility between the START-ART intervention and the sustainability period is shown in Fig. 2.

As shown in the figure, all health centers show no difference in ART initiation between the START-ART intervention period and the sustainability period. Although in some health centers the percentage decreased in the sustainability period, this was not statistically significant. Only one health center shows the intervention was not sustained (Fig. 2).

### Analysis of the trend of ART initiation within 14 days after eligibility between the START-ART intervention and the sustainability period at 6 months intervals from 2013 to 2016

**Fig. 3:**
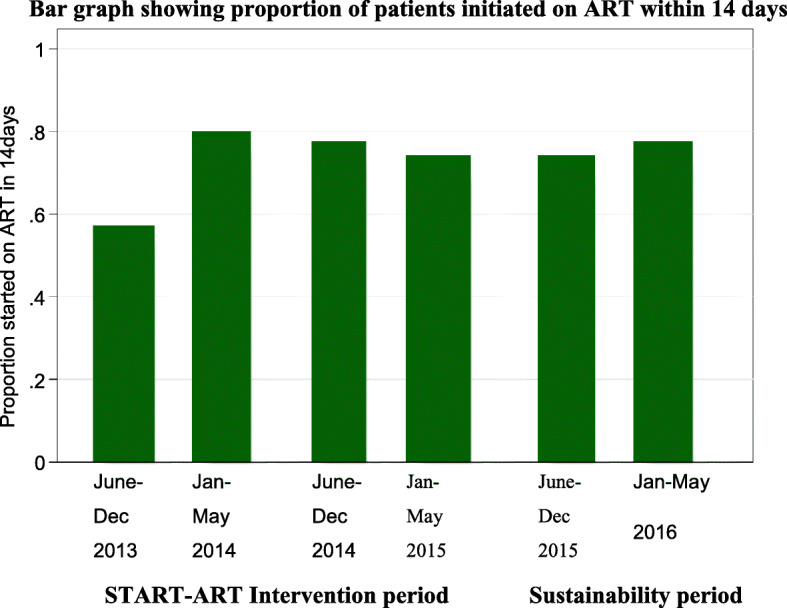
Health center analysis of ART initiation within 14 days after eligibility between the START-ART intervention and the sustainability period

Figure 3 shows a bar graph demonstrating a trend at 6 months intervals of ART initiation within 14 days from the START-ART intervention to the sustainability periods. As shown in the figure, there were no observable difference in the proportion of patients initiated on ART with 14 days of documented eligibility between the START-ART intervention and sustainability periods (Fig. 3)

### Facilitators to sustainability of START-ART in selected health centers

All health centers continued to initiate many PLHIV on ART within 14 days of eligibility after the end of the START-ART implementation intervention. However, there is variation in the proportion of PLHIV initiated on ART within 14 days. The highest-performing health center initiated 95.7% of eligible patients within 14 days, while the lowest-performing health center initiated 60.9% of the patients initiated within 14 days of documented eligibility.

During key informant interviews, health care providers identified facilitators to sustaining implementation of the START-ART intervention in participating health facilities (Table 3). Providers emphasized three facilitators: (1) understanding the benefits of early ART initiation; (2) new knowledge and skills imparted by the START-ART implementation, which led to its acceptability among health care workers; and (3) the physical opportunity to carry out early ART initiation provided by the POC PIMA machine, which avails CD4 cells results within 20 min.

**Table 3:** A Summary of factors that contributed to sustainability in the selected health center

Behavioral determinants	Facilitators to sustainability
**Capability** 1. Psychological 2. Physical	• Understood very well about the benefits of early ART initiation • The study provided knew knowledge and skills which they were willing to implement • None • Availability of the Pima machine that would avail CD4 counts within 20 min • None • None • None
**Opportunity** 1. Physical 2. Social	
**Motivation** 1. Automatic 2. Reflective	

Key informants emphasize that the physical opportunity offered by the system-strengthening activities, such as the provision of the POC CD4 PIMA machines to the health centers during the STARTS-ART implementation study, facilitated the sustainability of early ART initiation even after the study came to an end:

What changed is that before, when a patient came to test and they turned HIV positive, they would ask them to go and come back for their CD4 results. But when STARTS came, they brought the PIMA machine, so when a patient turned HIV positive, they would test his CD4s and results would be out immediately and patient starts drugs. That was continued. (KI interview, Peer Educator, Sustaining facility)

Key informants also pointed to the motivation of providing new skills and knowledge, so the health centers were eager to continue practicing what they had learned.

Studying doesn’t end, each time, you learn something new and you want to implement it (KI interview, clinical Officer, In-charge, Sustaining facility)

### Barriers to sustainability of START-ART in selected health centers

As shown in Table 4, health care workers identified three main barriers to sustaining the START-ART implementation: (1) lack of social opportunity capability to sustain due to lack of knowledge about the intervention, which was attributed to a lack of continuous mentorship as had been provided during the intervention implementation; (2) lack of physical opportunity to sustain early initiation due to expiry or stock outs of PIMA cartridges at the health centers; and (3) absence of supportive supervision, which respondents reported to decrease motivation (Table 4). Only one health center performed more poorly during the sustainability period than what was observed during the START-ART implementation study.

**Table 4:** A summary of barriers to sustainability in the selected health center

Behavioral determinants	Barriers to sustainability
**Capability** 1. Psychological 2. Physical	• Lack of knowledge about the intervention • No continuous training for the staff about the intervention • Newly transferred to the center without having acquired the skills previously trained by the STARTS-ART implementing team in the new health center • None • Pima cartridges would expire/stock out with no replacements • None • Health care providers attributed their more relaxed approach in the way they carried out their duties to staff transfers from one facility to another • Absence of support supervision which was previously provided by the study team • None
**Opportunity** 1. Physical 2. Social	
**Motivation** 1. Automatic 2. Reflective	

Health workers at this facility attributed low uptake of the intervention innovations during the sustainability period to the absence of continuous support from study staff. However, key informants at the facility that did sustain the intervention also reported a lack of supervision:

When they were implementing that program, it was ok. But when it ended, there has not been supervision since. (KI interview, ART counselor, unsustaining facility)

Health care workers also identified transfers made by their employer (district local government) from one health center to another as a key barrier to the sustainability of the START-ART practices. Previously, transfers were less common and health workers assumed that their place of assignment would be permanent. But a new policy introduced by the district local government led to staff transfers, which affected their motivation because these transfers were made with in the same district where START-ART implementation took place. It is believed they had received training in the implementation from their previous place of work.

There is change of staff and most people that are now at this facility don’t order for it. We have a laboratory technician, but there are no orders. It can only be used when there are requests. I think there has been a laxity. (KI interview, clinical Officer, In-charge, unsustaining facility)

Some components of the START-ART implementation required constant management and oversight to provide the physical opportunity for health care workers to initiate patients on ART within 14 days. For example, during the intervention period, the study team ensured that supplies were always available. During the sustainability period, however, facilities experienced stock-outs or expiry of PIMA cartridges, which stalled rapid assessment for ART eligibility.

You see we usually have problems with the PIMA, sometimes the cartridges expire or get finished, so we use it on and off. (KI interview, Laboratory technician, sustaining facility)

## Discussion

This mixed-methods study aimed to evaluate the sustainability of the rapid ART initiation and to identify the facilitators and barriers to sustaining rapid ART initiation among public sector health centers that participated in the START-ART implementation study. A greater proportion of eligible patients were initiated on ART within 2 weeks of eligibility during the sustainability period compared to the START-ART implementation intervention period (88.2% vs. 77.4%). The sustainability of implementing this intervention may have been enhanced because it was conducted within an HIV program, the Makerere University Joint AIDS Program. After the intervention period ended, MJAP continued to provide technical oversight for comprehensive HIV service delivery to the health centers that participated in the study [23]. This emphasizes the need for implementation research studies to engage with partners that are likely to continue implementing the intervention after the research study is over. This has also been shown by other studies which have been done in health care settings that are different from our study which also were well sustained beyond the study implementation through the system of engaging partners in implementing the intervention [24]. Sustaining an intervention depends on the value of the outcome associated with it [25] and engagement with partners that are likely to continue its implementation after the research study is over [24]. Each of these elements was present in the implementation of the START-ART intervention. The higher proportion of patients initiated on ART with 14 days in the sustainability period than during the period of active implementation could be attributed to the design of the START-ART implementation study. The stepped-wedge design meant that not all the health centers contributed equal amount of time in the START-ART intervention while in the sustainability study period, the time in the study was the same [12].

Notably, there were variations in the sustainability outcomes among the 15 health centers included in the present study, three of which performed significantly better in the sustainability period than the START-ART implementation study, while one health center performed worse during the sustainability period. Health workers who participated in key informant interviews explained these variations in terms of differences in motivation and consistent access to resources necessary for rapid ART initiation.

Key informants at participating health centers argued that the knowledge, skills, and appreciation for early ART initiation they gained during the START-ART implementation helped to sustain the practice of early ART initiation long after the study concluded. The START-ART implementation also provided a physical opportunity by providing POC PIMA machine which avails CD4 cells results within 20 min.

Our findings are similar to those reported by other authors, who have reported that scaling and sustaining an intervention requires proper training of the people who are going to implement the intervention, regularly supervising the implementers, motivating them, and ensuring adequate financial support, acceptability among community members, and support from political leaders and health care providers [26, 27]. In our study, health workers reported that the presence of the POC CD4 cell count PIMA analyzer encouraged them to initiate ART as per the ART initiation guidelines as soon as they got the patients’ CD4 results. These devices were handed over to the health centers, which motivated health workers.

On the other hand, the health care workers in the health center that performed worse in the sustainability period identified interrelated reasons for failure to sustain the gains made during the intervention period: (1) lack of supervision at the level provided during the START-ART implementation study, (2) replacement of old staff with new ones who were not well oriented onto the study components, and (3) stock-outs or lack of consistent PIMA cartridge supplies. During the implementation of the START-ART intervention, the study team conducted refresher trainings every 6 months and whenever required to remind the health care workers about intervention which was rapid ART initiation and its benefits. This was done during the feedback sessions where a report about the progress of the intervention in that specific health center would be made. However, after the end of the study, these trainings ended which could have affected the sustainability of the intervention [28].

Health workers at the facility that was unable to sustain the intervention cited staff transfers as a barrier to carrying out rapid ART initiation. In contrast, the health centers where the START-ART intervention was sustained had no staff transfers. Therefore, it was possible that some well-trained health care workers who understood START-ART intervention were transferred to other health centers affecting sustainability, as has been similarly reported by other authors [29, 30].

Our study had some strengths; the use of a mixed-methods approach gives our analysis both breadth and depth. The quantitative component allowed for a representative sample of health centers that participated in the START-ART intervention and patients eligible for early ART initiation, enabling us to assess the intervention’s sustainability. The qualitative component of the study enabled us to explore the facilitators to the sustainability of the intervention in the health centers where it was sustained and perceived barriers in the health center where it was not sustained.

The study also had some limitations. We excluded centers of excellence for HIV care which participated in the START-ART intervention because we thought they were different from the general health centers where most patients receive ART in rural Uganda. The centers of excellence are better resourced in terms of supplies and human resources capacity. By leaving them out, we could have underestimated or overestimated the sustainability of the intervention. This study was carried out, in the period when WHO had passed new guidelines for ART initiation of “test and treat” [8]. Uganda as a country had not yet adopted these new guidelines, and this was also explored during the KI interviews to ascertain when the health workers started rolling out the test and treat ART guidelines. This was found not to have had an effect as these guidelines were rolled a year later after the sustainability study [31].

In addition, qualitative data involved recalling what had been done in the preceding 2 years, so there might have been recall bias in the response to the questions. The health workers who were present during the START-ART intervention were more likely to respond positively to the questions during the KI by answering them in a way that would appear that they continued implementing the intervention even if they did not after the study it was over. Another limitation is that the sustainability analysis period began 2 months after the closure of the START-ART intervention study. It is therefore difficult to distinguish between the effects from the START-ART intervention study and the sustainability of the START-ART implementation study. However, because this effect persisted throughout the sustainability period, it less likely that this introduced bias. Finally, we selected only two sites for the qualitative analysis of facilitators and barriers to sustainability. However, since the key informants expressed broad consensus regarding facilitators and barriers, we believe that thematic saturation was achieved

## Conclusion

More patients were initiated on ART within 2 weeks of eligibility at facilities that had participated in the START-ART implementation intervention in the year after completion of the study than during the initial study period, suggesting that the intervention was robustly sustained. Integration of implementation science study methodologies within routine HIV care settings promotes sustainability of positive intervention.

## Data Availability

The datasets generated during and/or analyzed during the current study are not publicly available due ethical reasons but are available from the corresponding author on reasonable request.

## Abbreviations

AIDS: Acquired immunodeficiency syndrome
ARD: Adjusted risk difference
ART: Antiretroviral therapy
CD: Cluster of differentiation
COM-B: Capability, opportunity and motivation for behavior change
COREQ: Consolidated Criteria for Reporting Qualitative Research
HC: Health center
HIV: Human immunodeficiency virus
ID: Patient identification number
KI: Key informant interviews
MOH: Ministry of Health
OI: Opportunistic infection
PRECEDE: Predisposing, Reinforcing, and Enabling Constructs in Educational Diagnosis and Evaluation
POC: Point of care
PLHIV: People living with HIV/AIDS
RCT: Routine counseling and testing
RD: Risk difference
START-ART: Streamlined antiretroviral therapy initiation
UNAIDS: The Joint United Nations Programme on HIV/AIDS
WHO: World Health Organization
